# STAT3 Signaling Mediates Agomelatine Restoration of Prefrontal Cortex Synaptic Plasticity in Chronic Social Defeat Stress Mice

**DOI:** 10.1007/s12035-025-05638-2

**Published:** 2025-12-22

**Authors:** Chi-Wei Lee, Han-Fang Wu, Hsin-Ju Yen, Lun-De Liao, Wei-Chang Mao, Hsun-Shuo Chang, Yih-Fung Chen, Chia-Hsien Lin, Cheng-Ta Li, Hui-Ching Lin

**Affiliations:** 1 Brain Research Center, National Yang Ming Chiao Tung University, Taipei, Taiwan; 2 Department and Institute of Physiology, College of Medicine, National Yang Ming Chiao Tung University, Taipei, Taiwan; 3 Department of Optometry, MacKay Medical University, New Taipei City, Taiwan; 4 Institute of Biomedical Engineering and Nanomedicine, National Health Research Institute, Miaoli, Taiwan; 5 Department of Psychiatry, Cheng-Hsin General Hospital, Taipei, Taiwan; 6 School of Pharmacy, College of Pharmacy, Kaohsiung Medical University, Kaohsiung, Taiwan; 7 Department of Health Industry Management, Kainan University, Taoyuan, Taiwan; 8 Department of Psychiatry, Taipei Veterans General Hospital, No. 201., Sec. 2, Shih-Pai Road, Taipei, Taiwan; 9 Institute of Brain Science, National Yang Ming Chiao Tung University, Taipei, Taiwan; 10 Division of Psychiatry, Faculty of Medicine, National Yang Ming Chiao Tung University, Taipei, Taiwan; 11 Membrane Protein Structural Biology Research Center, National Yang Ming Chiao Tung University, Taipei, Taiwan; 12 Ph.D. Program in Medical Neuroscience, College of Medical Science and Technology, Taipei Medical University and National Health Research Institute, Taipei, Taiwan

**Keywords:** Chronic social defeat stress, Depression, Prefrontal cortex, STAT3, Agomelatine

## Abstract

Major depressive disorder (MDD) is a major public health issue worldwide. It not only causes an increased socio-economic burden, but also negatively affects personal lives. It has been shown that depression leads to impairments in synaptic plasticity, such as impaired long-term potentiation (LTP) in the prefrontal cortex (PFC). A recent study reported that administration of agomelatine, a melatonin receptor agonist and 5-hydroxytryptamine (5-HT) 2C antagonist, which may be involved in synaptic functions, for 1 week has a rapid antidepressive effect. Moreover, the mechanism of Glycogen synthase kinase 3β (GSK3β), regulated by signal transducer and activator of transcription 3 (STAT3), may play an important role in the synapse-related pathological mechanisms of depression. Hence, we used chronic social defeat stress (CSDS) as an animal model of depression, to investigate whether synaptic plasticity impairment in depression, improved by agomelatine, is mediated through the STAT3 mechanism. The results showed that depressive-like behaviors were reversed after 1 week of agomelatine treatment. Moreover, the impairment of LTP and alteration of spine density were reversed by agomelatine in the PFC. We further applied AG490, an inhibitor of STAT3, to demonstrate that the therapeutic mechanism of agomelatine improves synaptic plasticity through STAT3-regulated phosphorylation of GSK3β. Taken together, these results demonstrated that STAT3-regulated downstream molecules are potential therapeutic targets for the rapid action of antidepressants.

**Supplementary Information:**

The online version contains supplementary material available at 10.1007/s12035-025-05638-2.

## Introduction

MDD is a mental disorder that affects patients’ daily lives and is a major contributor to the worldwide burden of disease [1]. Symptoms of MDD include depressed mood, anhedonia, sleep disturbance, and suicidal thoughts [2]. The prefrontal cortex (PFC), a region of the brain that regulates thought and emotion in the corticolimbic network, is the area most impacted by depression [3–5]. A positron emission tomography (PET) imaging study demonstrated that decreased PFC glucose metabolism was significantly correlated with MDD [6]. An animal study showed that stress-induced depression reduced glutamate-mediated neurotransmission in the PFC [7]. Moreover, impairment in synaptic plasticity, including LTP in the PFC, has been implicated in depression animal [8–10]. Dendritic atrophy and spine loss have also been observed in animal models of depression [11, 12]. Further, previous studies have shown the α-amino-3-hydroxy-5-methyl-4-isoxazolepropionic acid (AMPA) receptors and postsynaptic protein postsynaptic density-95 (PSD-95) were decreased in animals and patients with MDD [13–15]. This suggests that glutamate-mediated synaptic function plays a critical role in depression.

At present, the majority of commonly prescribed antidepressants continue to target the monoaminergic system. These include selective serotonin reuptake inhibitors (SSRIs), dopamine–norepinephrine reuptake inhibitors, serotonin–norepinephrine reuptake inhibitors (SNRIs), and noradrenergic and specific serotonergic antagonists. All of these treatments are grounded in the monoamine hypothesis [16]. However, lower response and higher relapse rates remain a challenge in treating MDD [17, 18]. Moreover, an epidemiological study demonstrated that sleep disturbance is an important factor in the pathophysiology of MDD [19]. This suggests that new classes of antidepressants may play a key role in correcting the underlying circadian rhythm abnormalities in patients with MDD. Melatonin is an indolamine hormone secreted by the pineal gland [20]. A previous study showed that melatonin has synergistic effects when administered adjunctively with antidepressants [21]. Further, it has been reported that melatonin triggers the signal transducer and activator of transcription 3 (STAT3) [22]. The phosphorylated STAT3 acts on Glycogen synthase kinase 3β (GSK3β) to reduce its activity. This plays an important role in the antidepressant effects that have been demonstrated in chronic unpredictable mild stress (CUMS) rat model [23, 24]. In addition, previous studies have shown that stress-induced LTP impairment was followed by GSK3β activation [25–27].

To improve the weak antidepressive effects of melatonin, a new synthetic analog of melatonin, agomelatine, has been developed [28, 29]. In February 2009, the European Medicines Agency (EMA) approved agomelatine for the treatment of MDD in adults. Agomelatine, a 5-HT2C receptor antagonist and agonist of the melatonergic MT1 and MT2 receptors, acts as an antidepressant and its antidepressive effects have been shown in both animal and human studies [30, 31]. The study reported that stress-induced abnormal glutamate release could be reversed by agomelatine [32]. Short-term administration of agomelatine (7–10 days) has been shown to alleviate depressive-like behaviors in rodent models of depression, including improvements in behavioral deficits induced by chronic stress paradigms [33, 34]. Thus, the fast onset of agomelatine’s antidepressant action may result from the regulation of glutamate-related functions. However, the effect of agomelatine on the glutamatergic system in alleviating depressive-like symptoms remains unknown.

In the present study, we used a chronic social defeat stress (CSDS) mouse model to examine whether the antidepressant effects of agomelatine involve synaptic function. We further examined whether the molecular mechanisms of agomelatine, which improve depressive-like behavior and synaptic plasticity, are mediated by STAT3, which may regulate downstream effectors such as GSK3β. To further investigate whether the effects of agomelatine on depressive-like behavior and synaptic plasticity are mediated through STAT3, we employed AG490, a selective Janus kinase 2 (JAK2)/STAT3 inhibitor that has been shown to block STAT3 tyrosine phosphorylation and downstream signaling [35].

## Materials and Methods

### Animals

All animal experiments were conducted in accordance with the guidelines approved by the Institutional Animal Care and Use Committee (IACUC) of the College of Medicine at National Yang Ming Chiao Tung University (Taipei, Taiwan). Male C57BL/6 mice, 8 weeks of age, were used for all experiments. Animals were group-housed (4 to 5 per cage) in a controlled environment maintained at 24 °C, under a 12-h light/12-h dark cycle, with lights on starting at 7:00 AM. Standard rodent chow and water were provided ad libitum. All experimental procedures were carried out during the light phase of the cycle.

### Chronic Social Defeat Stress

C57BL/6 mice were subjected to the chronic social defeat stress (CSDS) paradigm, consisting of two daily social defeat sessions (5 min each) for a total of 10 consecutive days, following protocols previously established [36, 37]. In each session, the C57BL/6 mouse was placed into the home cage of a novel, aggressive CD1 retired breeder mouse (aged 4–6 months; Lasco Co) to induce physical confrontation. Because retired CD1 breeders are used as aggressors, only male C57BL/6 mice were included in this experiment to ensure consistent social defeat stress exposure. Each 5-min session was structured into two distinct phases: during the first 20 s (starting from the initial physical contact), direct interaction was allowed between the C57BL/6 and CD1 mice. For the remaining 280 s, the C57BL/6 mouse was placed inside a protective mesh cage within the CD1 mouse's home cage, allowing sensory but not physical contact. After the final defeat session, a social interaction test was conducted. This test consisted of two 5-min trials: one without a social target (target absent) and one with a CD1 mouse placed inside a small wire cage in the interaction zone (target present). The time spent by the C57BL/6 mouse in the defined interaction zone during each trial was recorded using a digital video camera and analyzed using SMART tracking software (version 3.0; Panlab, S.L.U., Spain). The interaction ratio was determined by dividing the time spent in the interaction zone during the target-present trial by the time spent during the target-absent trial. The interaction zone was defined as the 8-cm-wide area immediately surrounding the social target chamber, whereas each corner zone was defined as a 10 × 10 cm area located at the two corners opposite to the social target chamber. Mice with an interaction ratio less than 1 were classified as susceptible, while those with a ratio equal to or greater than 1 were considered resistant. Mice in the non-CSDS population also underwent the social interaction test. After the test, they were randomly assigned to either the non-CSDS group or the non-CSDS vehicle-treated group. The susceptible subpopulation, on the other hand, was randomly divided into four groups: untreated (hereafter referred to as “susceptible”), vehicle-treated, agomelatine-treated, and agomelatine combined with AG490-treated. Following the behavioral assessments, animals from each group were further randomly assigned for electrophysiology recording, Golgi staining, Western blot, and real-time PCR analyses, and were euthanized the day after the final behavioral test.

### Drug Application

Agomelatine and AG490 (Cayman Chemical) were freshly prepared on the day of use. Both compounds were dissolved in a vehicle consisting of 5% (v/v) DMSO in 0.9% saline to obtain a final working concentration of 1 mg/mL. Briefly, 10 mg of each compound was first dissolved in 500 µL DMSO and subsequently diluted with 9.5 mL saline to reach the final concentration (1 mg/mL, 5% DMSO). For drug administration, mice were divided into three groups: vehicle, agomelatine, and agomelatine combined with AG490. The vehicle group received the vehicle solution (5% DMSO in 0.9% saline), while treatment groups received agomelatine alone or agomelatine combined with AG490, with both compounds administered simultaneously at the indicated doses. All drugs were injected intraperitoneally at 10 mg/kg once daily for 1 week following CSDS induction. Based on a fixed injection volume of 10 mL/kg, the 1 mg/mL working solution delivered the target dose according to body weight (e.g., a 25 g mouse received 0.25 mL solution = 0.25 mg drug = 10 mg/kg).

### Tail Suspension Test (TST)

Mice were individually suspended by its tail from a horizontal ring-stand bar positioned 25 cm above the floor. Adhesive tape was applied approximately 1 cm from the tip of the tail. For 5 min video recorded, the immobility time was defined as the period during which the mouse remained completely still, exhibiting no active movements and hanging passively.

### Forced Swim Test (FST)

The FST was used to test the active coping behavior [38]. Mice were placed individually into a transparent cylinder (30 cm in diameter, 60 cm in height) filled with water to a depth of 30 cm, maintained at a temperature of 25 ± 1 °C. The test was conducted a 5-min period, during which the duration of immobility was recorded. Immobility was defined as the absence of any movement, with the mouse remaining completely still in the water.

### Sucrose Preference Test (SPT)

The sucrose preference test (SPT) was used to test for depression in the animals [39]. On the first day, immediately after completing the social interaction test, mice were habituated to the two-bottle choice paradigm to familiarize them with consuming water. At the start of the experiment, each mouse was housed individually in a test cage that similar with their home cage, and water was withheld for 2 h. During the testing phase, the mice were given access to two bottles: one containing a 2.5% sucrose solution and the other containing plain water. The testing period lasted for 4 h. The sucrose preference was calculated by dividing the volume of the sucrose solution consumed by the total amount of liquid consumed.

### Brain Slice Preparation and Electrophysiology Recording

After the behavioral tests, the mice were subsequently used for electrophysiological recordings. The mice were sacrificed by rapid decapitation. The brains were removed and placed in a beaker containing cold (4 °C) oxygenated (saturated with 95% O2 and 5% CO2) artificial cerebrospinal fluid solution (ACSF), consisting of (in mM) 117 mM NaCl, 4.7 mM KCl, 1.2 mM MgCl2, 1.2 mM NaHCO3, 2.5 mM CaCl, 25 mM NaHCO3, and 11 mM glucose. The brains were sectioned into 400 μm thick coronal slices. To measure the field excitatory postsynaptic potential (fEPSP) in the PFC, a concentric bipolar stimulating electrode (FHC; Bowdoinham, ME, USA) was positioned in layer II, while a capillary glass recording electrode (Harvard Apparatus) filled with 3 M NaCl solution was placed in layer V. The stimulation intensity was calibrated to produce a synaptic response at approximately half-maximal amplitude. Long-term potentiation (LTP) was induced through high-frequency stimulation (100 Hz for 1 s, with a 20-s interval between stimuli). Data were analyzed using pClamp software (version 10.3; Axon Instruments).

### Golgi Staining

Brain samples were prepared and processed for Golgi staining using the SliceGolgi Kit (Bioenno SliceGolgi Kit; Bioenno Tech). Coronal sections with a thickness of 100 μm were obtained using a microslicer (DTK-1000; Dosaka, Kyoto, Japan). The sections were then incubated in the impregnation solution in the dark at room temperature for 9 days. Following this, the sections were stained for 8 min with the staining solution and post-stained for an additional 4 min. The stained sections were mounted onto gelatin-coated slides and cover-slipped using Permount mounting medium. PFC synapse images were captured using an Olympus BX63 microscope. The dendritic spine protrusion lengths were measured using ImageJ software.

### Western Blot Assay

The PFC tissues were carefully dissected and homogenized in lysis buffer composed of 1% Triton X-100, 0.1% SDS, 50 mM Tris–HCl (pH 7.5), 0.3 M sucrose, 5 mM EDTA, 2 mM sodium pyrophosphate, 1 mM sodium orthovanadate, and 1 mM phenylmethylsulfonyl fluoride, supplemented with a complete protease inhibitor cocktail. After sonication, the homogenates were centrifuged at 12,000 rpm for 30 min to separate the supernatants. Protein concentrations were determined using the Bradford assay, and equal amounts of protein were loaded onto 7.5% acrylamide SDS-PAGE gels for electrophoresis. Proteins were then transferred to Immobilon-P membranes (Millipore) and blocked with 5% nonfat dry milk for 1 h at room temperature. Western blotting was performed using the following primary antibodies: phosphorylation of STAT3 at Tyr705 (Cell Signaling Technology), STAT3 (Cell Signaling Technology), phosphorylation of GSK3β at Ser9 (Cell Signaling Technology), GSK3β (Cell Signaling Technology), GluA1 (Abcam), GluA2 (Millipore), PSD-95 (Cell Signaling Technology), β-actin (Abcam), and GAPDH (GeneTex). Membranes were incubated with the primary antibodies overnight at 4 °C, followed by HRP-conjugated secondary antibody incubation for 1 h at room temperature. For STAT3 and phospho-STAT3, both antibodies were detected on the same membrane, with the membrane first incubated with the phospho-STAT3 antibody, then stripped and re-detected with the total STAT3 antibody. Stripping was performed by incubating the membrane in stripping buffer (50 mM Tris–HCl, pH 6.8, 2% SDS, and 100 mM β-mercaptoethanol) for 15 min at 50 °C. For GSK3β and phospho-GSK3β, were analyzed on separate membranes, with β-actin used as housekeeping controls. GluA1 was quantified using β-actin as the housekeeping control, GluA2 was quantified using GAPDH as the housekeeping control, and PSD-95 was quantified using β-actin as the housekeeping control. All housekeeping proteins were confirmed to be stable across experimental groups, ensuring optimal signal quality and accurate normalization. Protein signals were visualized using ECL Plus detection reagent (PerkinElmer, Boston, MA). Films were exposed for varying durations to ensure accurate signal detection without saturation, and band intensities were quantified using densitometry. The relative optical densities of the protein bands were analyzed with ImageJ software. For quantification, protein levels were first normalized to internal control and then expressed as fold changes relative to non-CSDS samples.

### Real Time PCR

Total RNA was extracted from PFC tissues using TRIzol reagent (Sigma-Aldrich). For cDNA synthesis, 1 µg of total RNA was reverse-transcribed in a 20 µL reaction mixture containing First-Strand buffer, 0.5 µg oligo(dT) primers, 0.5 mM dNTP mix, and M-MLV reverse transcriptase (Thermo Fisher Scientific). Quantitative PCR was performed using SYBR Green Master Mix (PCR Biosystems) on a real-time PCR system. The relative mRNA expression of interleukin-6 (IL-6) was normalized to the geometric mean of two housekeeping genes, β-actin and GAPDH. Previous studies have also used this combination of reference genes for cytokine (e.g., IL‑6) quantification or stress‑related animal study [40, 41]. Data were analyzed using the 2^ − ΔΔCt method and values were normalized to the control group.

The primer sequences were as follows:

- IL-6 (forward) 5′-CAAAGCCAGAGTCCTTCAGAG-3′;
- IL-6 (reverse) 5′-GTCCTTAGCCACTCCTTCTG-3′;
- β-actin (forward) 5′-CTGTCCCTGTATGCCTCTG-3′;
- β-actin (reverse) 5′-ATGTCACGCACGATTTCC-3′.
- GAPDH (forward) 5′-GACAACTCACTCAAGATTGTCAG-3′;
- GAPDH (reverse) 5′-ATGGCATGGACTGTGGTCATGAG-3′.

### Statistical Analysis

Data were analyzed using GraphPad Prism version 6 (GraphPad Software, San Diego, CA, USA). Results are presented as mean ± standard error of the mean (SEM). To assess group differences, one-way analysis of variance (ANOVA) followed by Bonferroni’s post hoc test and Kruskal–Wallis test were employed. This statistical approach was applied to evaluate behavioral performance, electrophysiological data, dendritic spine density, and protein expression levels between non-CSDS and susceptible groups. A p-value of less than 0.05 was considered statistically significant.

## Results

### Clarification the Susceptible Mice in CSDS

To investigate the mechanism of the effects of agomelatine on depression, we used the CSDS mouse model in the present study.

**Fig. 1:**
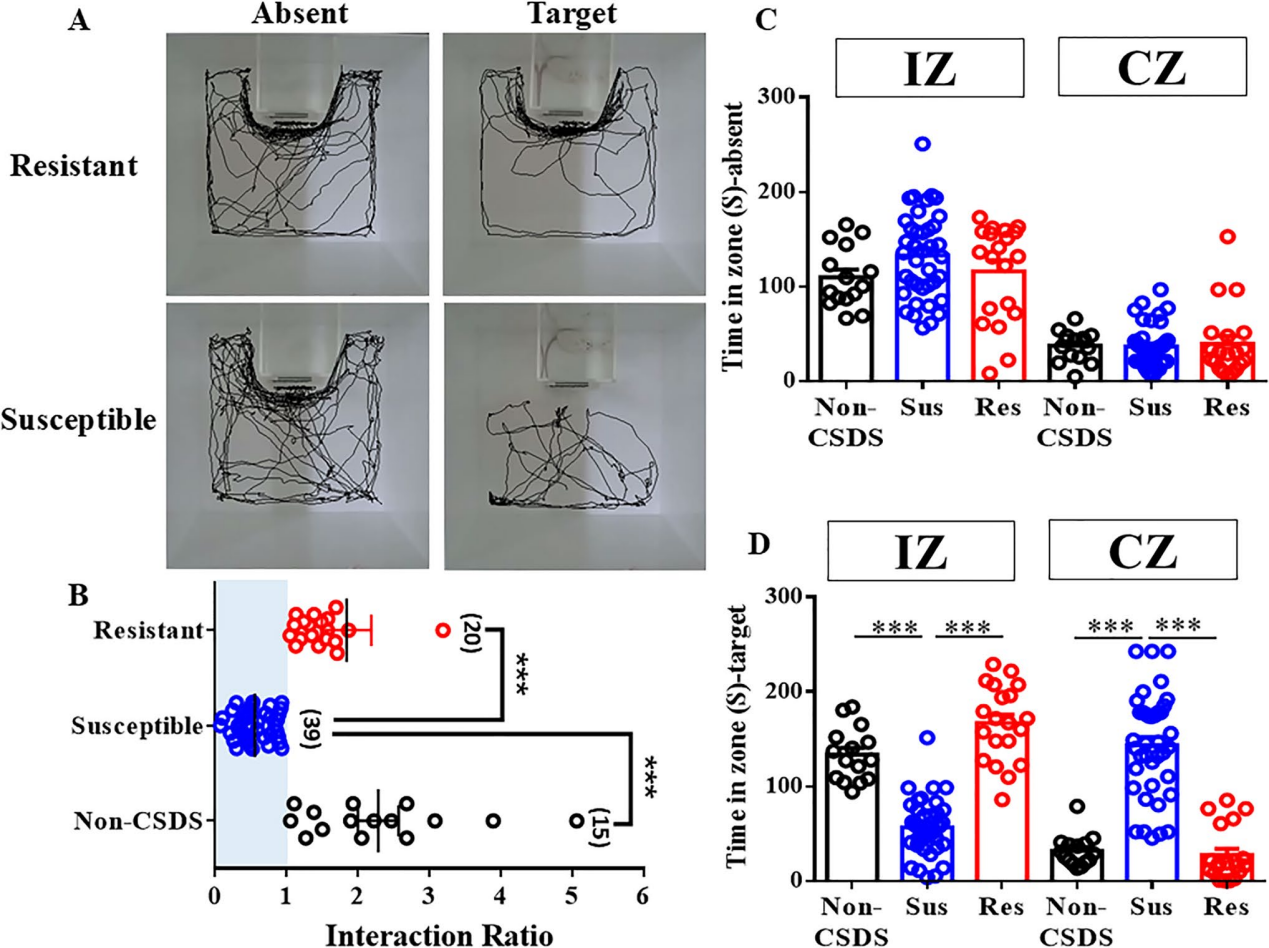
Mice submitted to CSDS could be separated into susceptible and resistant subpopulations. (**A**) The trace of resistant group and susceptible group movement during social interaction. (**B**) Horizontal scatter plot depicting the distribution of interaction ratio score for undefeated non-CSDS, susceptible (Sus) and resistant (Res) mice after a 10-day CSDS. (**C**) The time spent of non-CSDS, resistant and susceptible in the interaction zone (IZ) or corner zone (CZ) when the social target is absent. (**D**) The time spent of non-CSDS, resistant and susceptible in the interaction zone (IZ) or corner zone (CZ) when the social target is present. Data represent means ± SEM in each experiment. ***p < 0.001 versus susceptible group by one-way ANOVA (non-CSDS: *n* = 15, susceptible: *n* = 39, resistant: *n* = 20)

C57BL/6 mice were separated into susceptible and resistant subpopulations based on enduring deficits in social interactions (*F*_(2,71)_ = 22.85; Fig. 1A and 1B). The susceptible mice (0.5560 ± 0.040) displayed significant reduction in social interaction ratio compared with non-CSDS (2.286 ± 0.285, p < 0.001) and resistant (1.841 ± 0.346%, *p* < 0.001, Fig. 1B) mice. The time spent in the interaction zone and corner zone showed no significant difference between non-CSDS (*F*_(5,142)_ = 41.71; interaction zone: 109.7 ± 8.298s; corner zone: 37.08 ± 4.075s), resistant (interaction zone: 116.0 ± 11.34s; corner zone: 29.33 ± 8.232s), and susceptible (interaction zone: 132.5 ± 7.342s; corner zone: 36.39 ± 3.601s) mice during the absence of social targets (Fig. 1C). Otherwise, susceptible mice spent less time in the interaction zone (56.60 ± 4.720s) and more time in the corner zone (143.3 ± 8.708s) than the non-CSDS (*F*_(5,142)_ = 58.68; interaction zone: 133.4 ± 7.266s, *p* < 0.001; corner zone: 31.64 ± 4.245s, *p* < 0.001) and resistant (interaction zone: 166.8 ± 8.929s, *p* < 0.001; corner zone: 27.48 ± 6.306s, *p* < 0.001; Fig. 1D) mice during the target-present.

### Agomelatine Treatment Ameliorated Depressive-Like Behaviors and Active Coping Behavior in Susceptible Mice, which were Inhibited by AG490

**Fig. 2:**
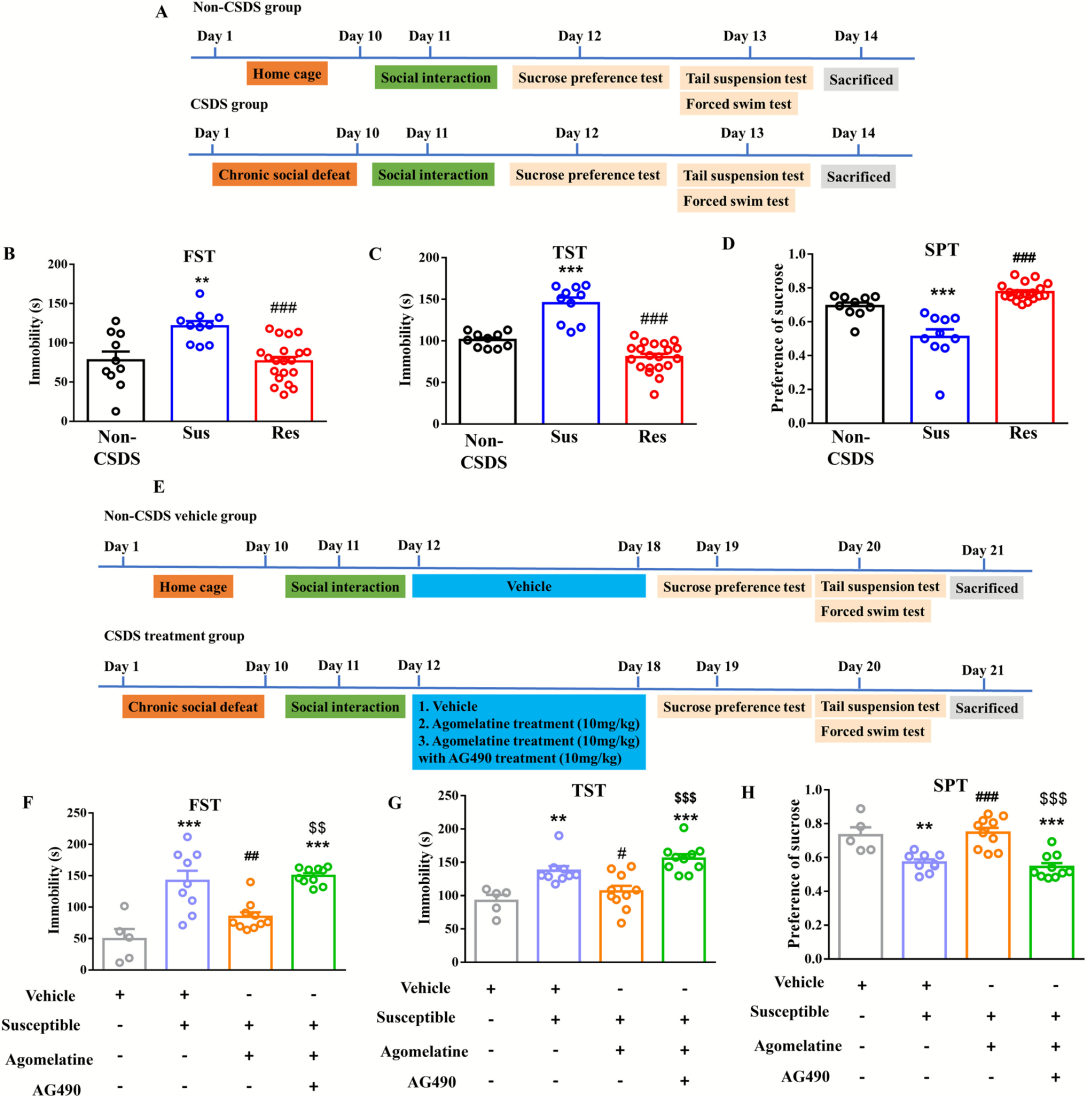
The depressive-like behavior in susceptible mice treating with agomelatine and combined with AG490. (**A**) The time line of experiment. (**B**) FST results in the non-CSDS, susceptible and resistant groups. Data represent means ± SEM in each experiment. **p < 0.01versus non-CSDS group, ###*p* < 0.001 versus susceptible group by one-way ANOVA. (**C**) TST results in the non-CSDS, susceptible and resistant groups. Data represent means ± SEM in each experiment. ***p < 0.001versus non-CSDS group, ###*p* < 0.001 versus susceptible group by one-way ANOVA. (**D**) SPT results in the non-CSDS, susceptible and resistant groups. Data represent means ± SEM in each experiment. ***p < 0.001versus non-CSDS group, ###p < 0.001 versus susceptible group by one-way ANOVA. (**E**) The time line of experiment in treatment group. (**F**) FST results in the non-CSDS vehicle, susceptible vehicle, agomelatine and combine with AG490. treatment groups. Data represent means ± SEM in each experiment. ***p < 0.001versus non-CSDS vehicle group, ##*p* < 0.01 versus susceptible vehicle group, $$*p* < 0.01 versus agomelatine group by one-way ANOVA. (**G**) TST results in the non-CSDS vehicle, susceptible vehicle, agomelatine and combine with AG490. treatment groups. Data represent means ± SEM in each experiment. **p < 0.01, ***p < 0.001 versus non-CSDS vehicle group, #*p* < 0.05 versus susceptible vehicle group, $$$*p* < 0.001 versus agomelatine group by one-way ANOVA. (H) SPT results in the non-CSDS vehicle, susceptible vehicle, agomelatine and combine with AG490. treatment groups. Data represent means ± SEM in each experiment. **p < 0.01, ***p < 0.001 versus non-CSDS vehicle group, ###*p* < 0.001 versus susceptible vehicle group, $$$*p* < 0.001 versus agomelatine group by one-way ANOVA. (non-CSDS: *n* = 10, non-CSDS vehicle: *n* = 5, resistant: *n* = 20, susceptible: *n* = 10, susceptible vehicle: *n* = 9, susceptible + agomelatine: *n* = 10, susceptible + agomelatine + AG490: *n* = 10)

After the social interaction test, mice were classified into non-CSDS, susceptible, and resistant groups and proceeded along the no-treatment timeline (Fig. 2A).

A separate cohort followed the drug-treatment timeline (Fig. 2E), consisting of non-CSDS vehicle-treated mice and CSDS mice treated with vehicle, agomelatine, or agomelatine plus AG490. Behavioral assessments were subsequently conducted according to each timeline. Following the social interaction test or drug treatment, mice first underwent the SPT, which included a same-day adaptation period to two bottles of water as part of the low-stress assessment of anhedonia. In contrast, the subsequent behavioral tests, the TST and the FST, did not include any habituation period. The TST was conducted the next day, followed by the FST after a 12-h interval, as recommended by Castagné et al. (2011), allowing assessment of behavioral depression while minimizing acute fatigue [42]. The FST results indicated that immobility duration was significantly increased in susceptible mice compared with non-CSDS mice (*F*_(2,37)_ = 9.857; non-CSDS: 77.73 ± 11.13s; susceptible: 121.1 ± 6.611s, *p* < 0.01 vs non-CSDS; Fig. 2B). The immobility duration was significantly decreased in resistant mice compared with susceptible mice (76.44 ± 5.706s, *p* < 0.001 vs susceptible; Fig. 2B). There was no significant difference between non-CSDS and resistant mice (*p* = 0.9919 vs non-CSDS; Fig. 2B). The TST results indicated that immobility duration was significantly increased in susceptible mice compared with non-CSDS mice (*F*_(2,37)_ = 47.19; non-CSDS: 101.1 ± 2.868s; susceptible: 145.2 ± 6.898s, *p* < 0.001 vs non-CSDS; Fig. 2C). The immobility duration was significantly decreased in resistant mice compared with susceptible mice (80.43 ± 3.953s, *p* < 0.001 vs susceptible; Fig. 2C). There was no significant difference between non-CSDS and resistant mice (*p* = 0.0701 vs non-CSDS; Fig. 2C). The SPT results showed that the sucrose preference was significantly decreased in susceptible mice compared with non-CSDS mice (*F*_(2,37)_ = 32.76; non-CSDS: 0.693 ± 0.021; susceptible: 0.5105 ± 0.044, *p* < 0.001 vs non-CSDS; Fig. 2D). The sucrose preference was significantly increased in resistant mice compared with susceptible mice (0.775 ± 0.011, *p* < 0.001 vs susceptible; Fig. 2D). There was no significant difference between non-CSDS and resistant mice (*p* = 0.0848 vs non-CSDS; Fig. 2D). Moreover, the immobility duration was also significantly increased in susceptible vehicle-treated mice compared with non-CSDS vehicle-treated mice in FST (*F*_(3,30)_ = 16.42; non-CSDS vehicle: 49.19 ± 16.17s; susceptible vehicle: 142.0 ± 16.09s *p* < 0.001 vs non-CSDS vehicle; Fig. 2F). Administration of agomelatine significantly reduced the immobility duration compared with susceptible vehicle-treated mice (84.72 ± 7.235s, *p* < 0.01 vs susceptible vehicle; Fig. 2F). This was inhibited by AG490 in FST (149.5 ± 4.190s, *p* < 0.001 vs non-CSDS vehicle; Fig. 2F). In addition, AG490 treatment showed a significant difference compared with agomelatine treatment (*p* < 0.01 vs agomelatine; Fig. 2F). Furthermore, the immobility duration was also significantly increased in susceptible vehicle-treated mice compared with non-CSDS vehicle-treated mice in TST (*F*_(3,30)_ = 12.46; non-CSDS vehicle: 92.29 ± 8.856s; susceptible vehicle: 137.3 ± 7.033s *p* < 0.01 vs non-CSDS vehicle; Fig. 2G). The immobility duration was significantly decreased by agomelatine treatment compared with susceptible vehicle-treated mice (106.3 ± 8.413s, *p* < 0.05 vs susceptible vehicle; Fig. 2G), and this was inhibited by AG490 in TST (155.5 ± 6.713s, *p* < 0.001 vs non-CSDS vehicle, *p* < 0.001 vs agomelatine; Fig. 2G). The sucrose preference was also significantly decreased in susceptible vehicle-treated mice compared with non-CSDS vehicle-treated mice (*F*_(3,30)_ = 15.50; non-CSDS vehicle: 0.7319 ± 0.047; susceptible vehicle: 0.5708 ± 0.018 *p* < 0.01 vs non-CSDS vehicle; Fig. 2H). The preference of sucrose was increased by agomelatine treatment compared with susceptible vehicle-treated mice (0.746 ± 0.028, *p* < 0.001 vs susceptible vehicle; Fig. 2H), and this was inhibited by AG490 (0.544 ± 0.022, *p* < 0.001 vs non-CSDS vehicle, *p* < 0.001 vs agomelatine; Fig. 2H), suggesting the agomelatine treatment improved depressive-like behaviors and active coping behavior were through the STAT3 signaling pathway.

### Impaired Long-term Potentiation in Susceptible Mice was Ameliorated by Agomelatine Treatment through the STAT3 Signaling Pathway

**Fig. 3:**
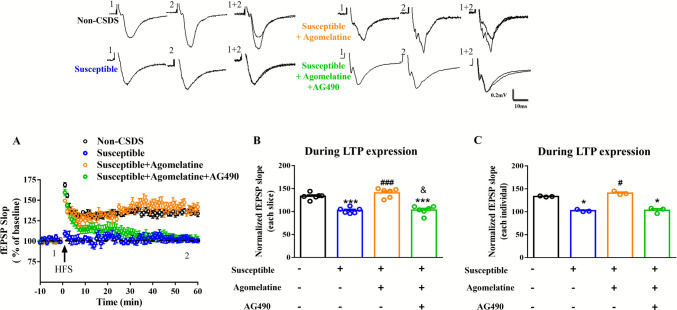
LTP recording from PFC of susceptible mice treating with agomelatine and combined with AG490. (**A**) The trace of each group in electrical physiology. (A) Recording of LTP induced by high-frequency stimulation (HFS). (**B**) The average fEPSP slope from each slice was calculated during the last 10 min. (**C**) The average fEPSP slope from each mouse was calculated during the last 10 min. Data represent means ± SEM in each experiment. *p < 0.05, ***p < 0.001 versus non-CSDS group, #*p* < 0.05, ###*p* < 0.001 versus susceptible group, &*p* < 0.05 versus agomelatine treated group by one-way ANOVA and Kruskal–Wallis test (each group *n* = 6 slice from 3 mice)

To confirm the effects of agomelatine on synaptic plasticity, we performed electrical physiology experiments. The results showed that the last 10 min response also indicated that the LTP was impaired in susceptible mice (*F*_(3,20)_ = 34.79; 102.4 ± 2.566%, *p* < 0.001 vs non-CSDS; Fig. 3 A and B). Moreover, agomelatine treatment improved the impaired LTP in susceptible mice (140.8 ± 4.124%, *p* < 0.001 vs susceptible), and this was inhibited by AG490 (103.2 ± 3.863%, *p* < 0.001 vs non-CSDS; Fig. 3A and B). In addition, a significant decrease was also observed after AG490 treatment compared with agomelatine treatment (*p* < 0.05 vs agomelatine; Fig. 3 B). We further analyzed the averaging all recorded slices from each individual animal. The results also indicating the agomelatine treatment improved the impaired LTP in susceptible mice (133.4 ± 0.675%, *p* < 0.05 vs susceptible), and this was inhibited by AG490 (103.2 ± 3.506%, *p* < 0.05 vs non-CSDS; Fig. 3C). However, there was no significant difference between AG490 treatment and agomelatine treatment (*p* = 0.1047 vs agomelatine; Fig. 3C). These results suggest that agomelatine improves LTP via the STAT3 signaling pathway.

### Decreased Dendritic Spine Density in Susceptible Mice was Improved by Agomelatine Treatment though the STAT3 Signaling Pathway

**Fig. 4:**
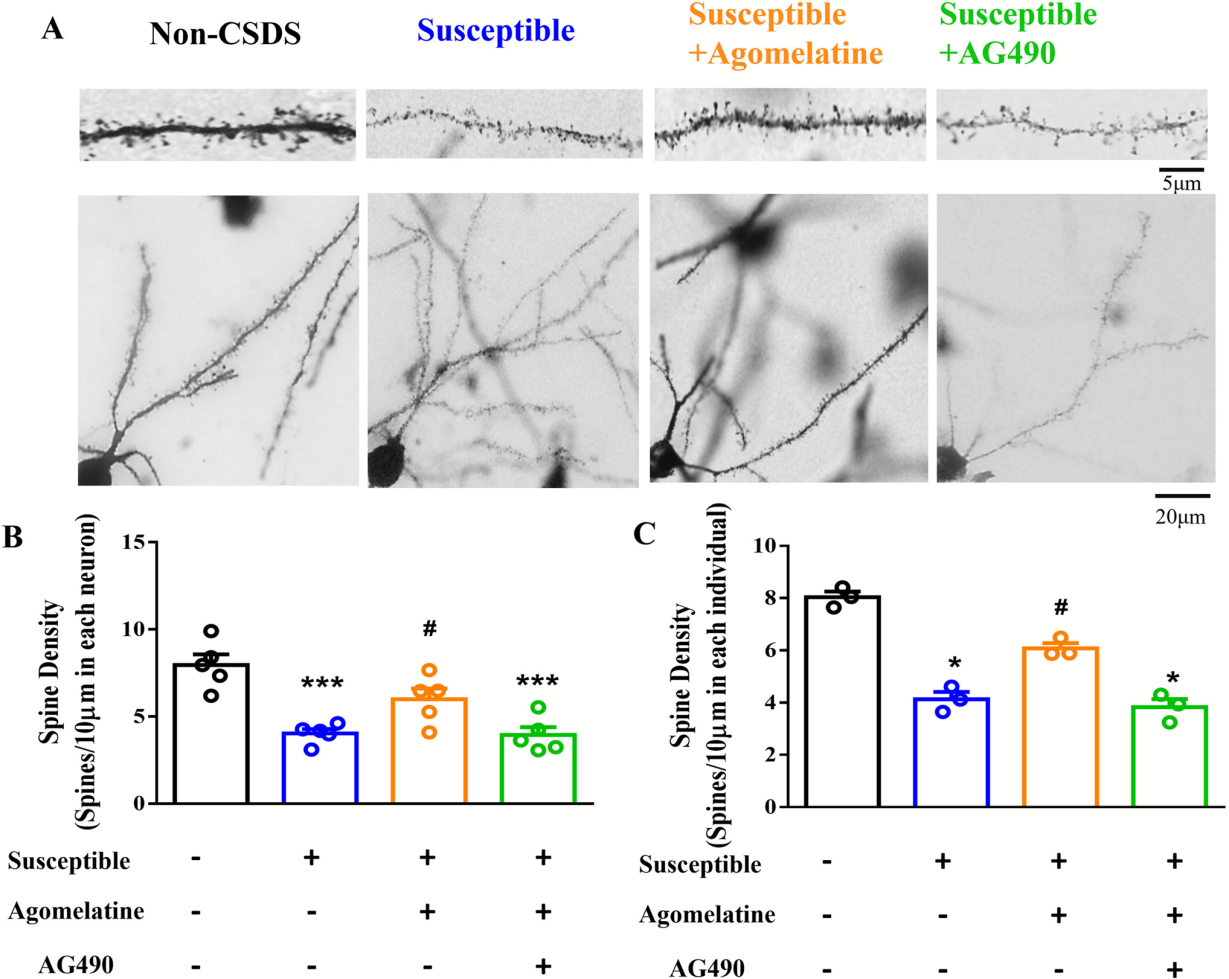
The density of dendritic spine in PFC of susceptible mice treating with agomelatine and combined with AG490. (**A**) Representative Golgi-stained sections of spines density in PFC (the upper scale bar indicated 5 μm; the lower scale bar indicated 20 μm). (**B**) Bar chart comparing the spine density for each neuron across groups. (**C**) Bar chart comparing the spine density for each mouse across groups. Data represent means ± SEM in each experiment. *p < 0.05, ***p < 0.001 versus non-CSDS group, #*p* < 0.05 versus susceptible group by one-way ANOVA and Kruskal–Wallis test (each group *n* = 5 neurons from 3 mice)

Next, we applied Golgi staining to assess the spine density in layer V of the PFC (Fig. 4A), and the results showed that the spine density was significantly decreased in susceptible mice as compared with the non-CSDS mice (*F*_(3,16)_ = 14.39; 4.031 ± 0.253 spines per 10 μm, *p* < 0.001 vs non-CSDS; Fig. 4B). The decreased spine density was improved by agomelatine treatment (5.997 ± 0.608 spines per 10 μm, *p* < 0.05 vs susceptible), and this was inhibited by AG490 (3.965 ± 0.444 spines per 10 μm, *p* < 0.001 vs non-CSDS; Fig. 4B). However, there was no significant difference between AG490 treatment and agomelatine treatment (*p* = 0.3686 vs agomelatine; Fig. 4B). We further analyzed the averaging all recorded neuron from each individual animal. The results also indicating the agomelatine treatment improved the decreased spine density in susceptible mice (6.079 ± 0.2026 spines per 10 μm, *p* < 0.05 vs susceptible), and this was inhibited by AG490 (3.828 ± 3.107 spines per 10 μm, *p* < 0.05 vs non-CSDS; Fig. 4C). There was no significant difference between AG490 treatment and agomelatine treatment (*p* = 0.5369 vs agomelatine; Fig. 4C). These findings suggest that agomelatine improves spine density through the STAT3 signaling pathway.

### Activation of STAT3 Signaling Pathway and Expression of Synaptic Protein were Improved by Agomelatine Treatment in Susceptible Mice

We examined whether the mechanism of action of agomelatine involves the STAT3 signaling pathway.

**Fig. 5:**
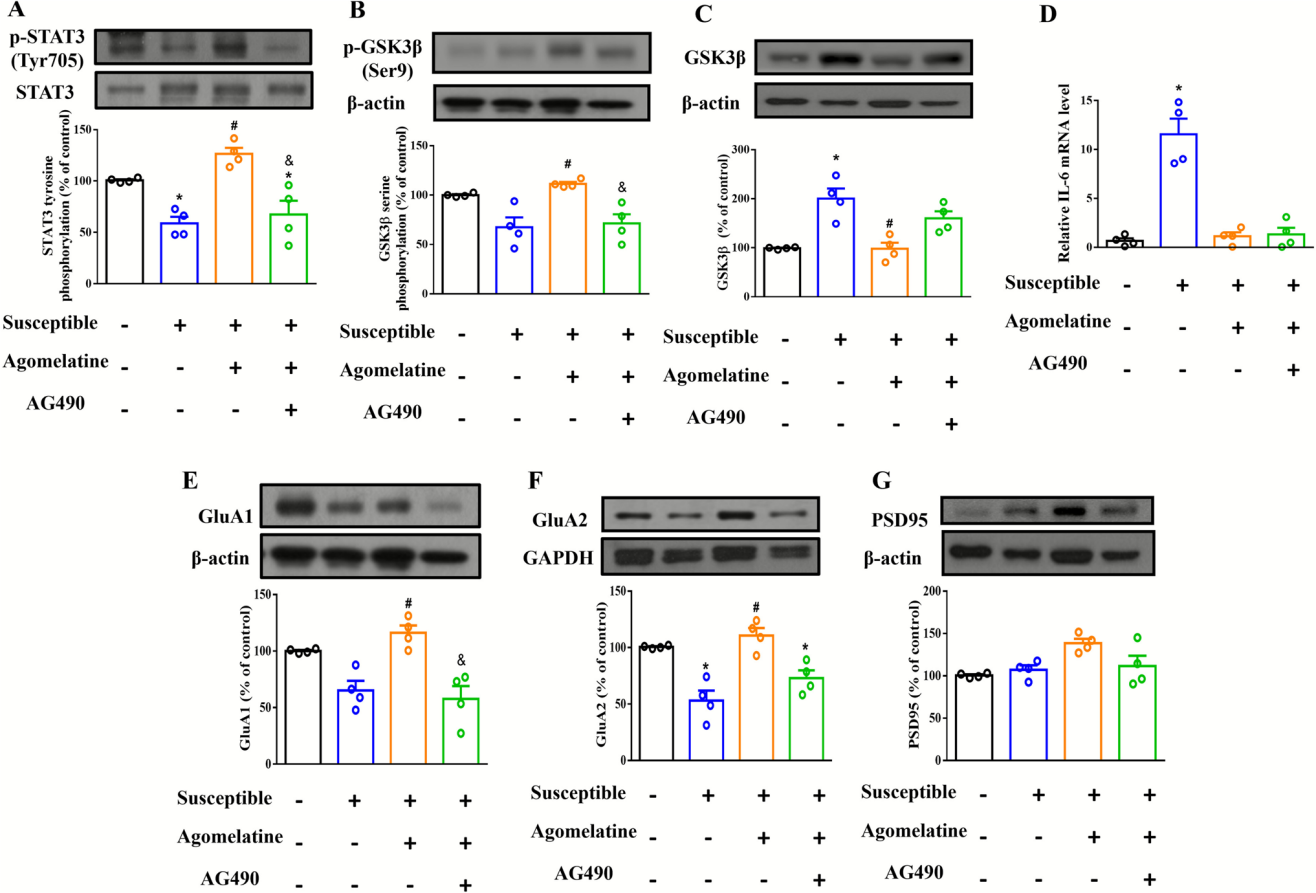
The STAT3 singling and synaptic protein expression in PFC of susceptible mice treating with agomelatine and combined with AG490. (**A**) Representative Western blot and summary bar graph of phosphorylation levels of STAT3 in the PFC. (**B**) Representative Western blot and summary bar graph of phosphorylation levels of GSK3β in the PFC. (**C**) Representative Western blot and summary bar graph of expression levels of GSK3β in the PFC. (**D**) Relative IL-6 mRNA expression normalized to the geometric mean of two housekeeping genes, β-actin and GAPDH in PFC (**E**) Representative Western blot and summary bar graph of expression levels of GluA1 in the PFC. (**F**) Representative Western blot and summary bar graph of expression levels of GluA2 in the PFC. (**G**) Representative Western blot and summary bar graph of expression levels of PSD95 in the PFC. Data are represented as the mean ± SEM. *p < 0.05 versus non-CSDS group, #*p* < 0.05 versus susceptible group, &*p* < 0.05 versus agomelatine treated group by Kruskal–Wallis test (each group *n* = 4)

The phosphorylation of STAT3 in the PFC was significantly decreased in susceptible mice (58.80 ± 6.376%, p < 0.05 vs non-CSDS; Fig. 5A). The decreased STAT3 phosphorylation was improved by agomelatine treatment (126.4 ± 5.957%, *p* < 0.05 vs susceptible), and inhibited by AG490 (67.48 ± 13.35%, *p* < 0.05 vs non-CSDS; Fig. 5A). There was the significant decreased in STAT3 phosphorylation after AG490 treatment compared with agomelatine treatment (67.48 ± 13.35%, *p* < 0.05 vs agomelatine; Fig. 5A). We also examined the housekeeping controls for both p-STAT3 and STAT3. Consistent with the previous results, phosphorylation of STAT3 in the PFC was significantly decreased in susceptible mice, restored by agomelatine treatment, and inhibited by AG490. Moreover, STAT3 phosphorylation was significantly lower in the AG490 group compared with the agomelatine group. In contrast, total STAT3 levels showed no significant difference among groups (Supplementary Fig. 1). Given that hyper‑activity of GSK3β is implicated in stress‑induced synaptic plasticity deficits and that phosphorylation changes may occur independently of total protein levels [43], this separation is particularly important because GSK3β activity is predominantly regulated through inhibitory phosphorylation at Ser9 rather than through changes in total protein abundance, as highlighted by Beurel et al. [44]. Therefore, we analyzed phosphorylated GSK3β and total GSK3β separately using housekeeping controls. There was a trend toward decreased phosphorylation of GSK3β in the PFC of susceptible mice, although the difference was not statistically significant (67.39 ± 10.00%, *p* = 0.3806 vs non-CSDS; Fig. 5B). The decrease in GSK3β phosphorylation was increased by agomelatine treatment (111.4 ± 1.925%, *p* < 0.05 vs susceptible), and decreased by AG490 (71.28 ± 9.229%, *p* = 0.5262 vs non-CSDS; Fig. 5B). The significant decreased in GSK3β phosphorylation after AG490 treatment compared with agomelatine treatment was observed (*p* < 0.05 vs agomelatine; Fig. 5B). Whereas, the expression of GSK3β in the PFC was significantly increased in susceptible mice (200.4 ± 20.46%, *p* < 0.05 vs non-CSDS; Fig. 5C). The increased GSK3β expression was decreased by agomelatine treatment (98.03 ± 12.32%, *p* < 0.05 vs susceptible), and decreased by AG490 (160.1 ± 14.25%, *p* = 0.3214 vs non-CSDS; Fig. 5C), however there was no significant difference between AG490 treatment and agomelatine treatment (*p* = 0.3214 vs agomelatine; Fig. 5C). We further examined the p-GSK3β/total GSK3β ratio, using independent membranes for phospho- and total-protein detection (Supplementary Fig. 2). Susceptible mice showed a trend of reduced GSK3β phosphorylation, which was significantly restored by agomelatine, indicating normalization of GSK3β activity via inhibitory phosphorylation independent of total protein levels. As previous study reported, STAT3 activation alongside elevated levels of its upstream regulator, IL-6 [45], we next sought to determine the expression of IL-6 mRNA level. The result indicated that the expression of IL-6 mRNA level in the PFC was significantly increased in susceptible mice (11.56 ± 1.604, *p* < 0.05 vs non-CSDS; Fig. 5D). The increased IL-6 mRNA level was decreased after agomelatine treatment, although the difference was not statistically significant (1.125 ± 0.408, *p* > 0.99 vs non-CSDS, *p* = 0.1556 vs susceptible; Fig. 5D). And there was no significant difference between AG490 treatment and either non-CSDS or agomelatine treatment (1.315 ± 0.683, *p* > 0.99 vs non-CSDS, *p* > 0.99 vs agomelatine; Fig. 5D). We further examined whether agomelatine treatment increased the expression of synaptic proteins. The results showed that the level of AMPA receptor GluA1 subunit in the PFC was decreased in the PFC of susceptible mice, although the difference was not statistically significant (65.22 ± 18.418%, *p* = 0.3806 vs non-CSDS; Fig. 5E). The decreased GluA1 expression was improved by agomelatine treatment (116.1 ± 6.563%, *p* < 0.05 vs susceptible), and decreased by AG490 (57.65 ± 11.36%, *p* = 0.2701 vs non-CSDS; Fig. 5E). There was the significant decreased in GluA1 after AG490 treatment compared with agomelatine treatment (*p* < 0.05 vs agomelatine; Fig. 5E). The level of AMPA receptor GluA2 subunit in the PFC was significantly decreased in susceptible mice (53.01 ± 8.944%, p < 0.05 vs non-CSDS; Fig. 5F). The decreased GluA2 expression was improved by agomelatine treatment (110.7 ± 6.661%, *p* < 0.05 vs susceptible). In contrast, it was inhibited by AG490 (72.98 ± 6.846%, *p* < 0.05 vs non-CSDS; Fig. 5F). There was no significant difference between AG490 treatment and agomelatine treatment (*p* = 0.1556 vs agomelatine; Fig. 5F). The expression of PSD 95 did not change in each group (non-CSDS: 100.4 ± 1.292%; susceptible: 107.1 ± 5.226%, *p* = 0.772 vs non-CSDS; agomelatine: 138.5 ± 5.226%, *p* = 5.299 vs susceptible; AG490: 111.6 ± 12.26%, *p* > 0.99 vs non-CSDS, *p* = 0.321 vs agomelatine; Fig. 5G).

## Discussion

The present study provides evidence that agomelatine administration improves depressive-like behaviors in CSDS mice. The impaired LTP and alteration of synaptic morphology were improved by agomelatine administration through STAT3 and GSK3β activation in CSDS mice.

In the present study, we employed the CSDS paradigm to investigate mechanism of agomelatine in treating depression. In our previous publication, we validated that classification based on the social interaction ratio effectively distinguishes resilient and susceptible population, with resilient mice showing no depressive-like behaviors and susceptible mice exhibiting clear depressive-like behaviors [46]. In the current study, we also validated this behavioral distinction, confirming that the classification criterion reliably reflects the depressive-like phenotype rather than an arbitrary cutoff around the threshold. Therefore, the behavioral classification was not determined by marginal differences near the social interaction ratio threshold (e.g., 0.95 vs. 1.05), but rather by consistent and statistically supported behavioral divergence between the resilient and susceptible groups. Moreover, our results showed that susceptible mice exhibited reduced STAT3 phosphorylation and increased GSK3β activity, accompanied by an elevation of IL-6, a key upstream regulator of STAT3 signaling. Indeed, a close association between STAT3 signaling, stress, and depression have been reported. Previous study demonstrated that intracerebroventricular administration of IL-6 induces depression-like behavior by activating STAT3-dependent regulation of serotonin transporter function, highlighting the interplay between IL-6 elevation and STAT3 signaling in mood dysregulation [47]. The restoration of miR-124 alleviated depressive-like behavior by inhibiting STAT3-mediated microglial activation and neuroinflammation was demonstrated by chronic unpredictable mild stress mice [48]. However, these findings differ from our observations, and previous research has indicated that STAT3 is involved not only in IL-6–related pathways but also in other mechanisms relevant to stress and depression. Other study indicated that STAT3 functions as a cytoprotective factor against oxidative stress, as its deficiency markedly increases susceptibility to apoptosis under hydrogen peroxide–induced oxidative stress [49]. In line with this, the CSDS induce depressive-like behavior and hippocampal oxidative stress, which can be ameliorated by agomelatine treatment has been reported [33]. Moreover, STAT3 overexpression was found to ameliorate hTau-associated synaptic deficits in an Alzheimer’s disease model [50], suggesting that STAT3 may influence depression through multiple, context-dependent mechanisms involving oxidative stress, synaptic regulation, and neuroinflammation.

Our results demonstrated that agomelatine treatment effectively alleviated CSDS-induced depressive-like behaviors, accompanied by increased STAT3 phosphorylation and decreased GSK3β activity. Furthermore, the antidepressant effects of agomelatine were confirmed to be mediated through STAT3 activation, as co-treatment with the STAT3 antagonist AG490 abolished these effects. Although IL-6 levels showed only a decreasing trend following agomelatine treatment, this reduction was not reversed by STAT3 inhibition. Previous studies have also demonstrated that the mechanism of agomelatine involves modulation of STAT3 and IL-6. In the chronic mild stress model, both phosphorylated STAT3 and IL-6 levels were elevated, and five weeks of agomelatine treatment significantly reduced these increases [51]. Interestingly, agomelatine administration alone was reported to enhance nuclear STAT3 phosphorylation [51], suggesting a context-dependent regulatory effect. In addition, agomelatine has been shown to increase cytosolic p-GSK3β levels in the PFC [52], further supporting that its antidepressant actions involve coordinated modulation of STAT3 and GSK3β phosphorylation.

Our previous study demonstrated the impairment of synaptic plasticity in the susceptible group after CSDS [46]. Indeed, aberrant spine morphology and synaptic plasticity, including LTP, long-term depression (LTD), and synaptic protein levels, have been observed in both depressed subjects and stressed animals [53]. The results of the present study indicate that the impairment of synaptic plasticity and decreased spine density in the PFC of CSDS mice were improved after 1 week of agomelatine treatment. A previous study indicated that long-term (20 weeks) agomelatine treatment improves cognitive performance, hippocampal spine density, and spine maturation in rats [54]. Another study demonstrated that agomelatine treatment improved CSDS-induced memory deficits and the mRNA expression of synaptic plasticity markers, including *PSD-95*, *Synaptophysin*, *Spinophilin* and *Synpasin I* genes in the hippocampus [55].

Furthermore, our results indicate that agomelatine improves synaptic plasticity, with changes in dendritic spine density being associated with STAT3 signaling, highlighting a potential mechanistic link between synaptic plasticity and STAT3-mediated pathways in the PFC of stressed mice. Overexpression of STAT3 improves LTP and spine density in the hippocampus in a mouse model of frontotemporal dementia [56]. An epilepsy study suggests that the STAT3 pathway regulates synaptic plasticity in epileptic mice [57]. Moreover, a focal ischemic study indicated that STAT3 directly binds to plasticity-related genes, including *BDNF*, *PICK1,* and *synaptophysin* [58].

The STAT3-modulated GSK3β activity’s antidepressant effects have been demonstrated in animal studies [23, 24]. The phosphorylation of STAT3 at Tyr705 inducing the phosphorylation of GSK3β at Ser9 leading to inactivation of GSK3β activity has been reported [59]. Our results also indicated that the antidepressant effect of agomelatine was through STAT3 phosphorylation contributing to the regulation GSK3β activity. Additionally, the activation of GSK3β also showed the important role in stress-induced LTP impairment [25], suggesting that the activation mechanism of GSK3β regulated by STAT3 plays a crucial role in the synaptic plasticity deficits caused by stress.

In conclusion, this study provides a novel antidepressant mechanism for agomelatine, STAT3-related downstream molecules regulated by agomelatine may become new therapeutic targets for the rapid treatment of depression.

## Supplementary Information

Below is the link to the electronic supplementary material.

Supplementary file1 (PDF 383 KB)

## Data Availability

No datasets were generated or analyzed during the current study.
