# Supplementary material for: STAT3 Signaling Mediates Agomelatine Restoration of Prefrontal Cortex Synaptic Plasticity in Chronic Social Defeat Stress Mice
Source: Mol Neurobiol. 2025 Dec 22;63(1):320. doi: 10.1007/s12035-025-05638-2 (PMC12722381; doi:10.1007/s12035-025-05638-2)
Supplement: Supplementary file 1 — Supplementary file1 (PDF 383 KB) [file 12035_2025_5638_MOESM1_ESM.pdf]

STAT3 signaling mediates agomelatine restoration of prefrontal cortex synaptic plasticity in chronic social defeat stress mice

Chi-Wei Lee[1,2], Han-Fang Wu[3], Hsin-Ju Yen[2], Lun-De Liao[4], Wei-Chang Mao[5], Hsun-Shuo Chang[6], Yih-Fung Chen[6], Chia-Hsien Lin[7], Cheng-Ta Li*[1,8,9,10], Hui-Ching Lin*[1,2,11,12]

[1] Brain Research Center, National Yang Ming Chiao Tung University, Taipei, Taiwan;

[2] Department and Institute of Physiology, College of Medicine, National Yang Ming Chiao Tung University, Taipei, Taiwan;

[3] Department of Optometry, MacKay Medical College, New Taipei City, Taiwan;

[4] Institute of Biomedical Engineering and Nanomedicine, National Health Research Institute, Miaoli, Taiwan;

[5] Department of Psychiatry, Cheng-Hsin General Hospital, Taipei, Taiwan;

[6] School of Pharmacy, College of Pharmacy, Kaohsiung Medical University, Kaohsiung, Taiwan;

[7] Department of Health Industry Management, Kainan University, Taoyuan, Taiwan;

[8] Department of Psychiatry, Taipei Veterans General Hospital, Taipei, Taiwan;

[9] Institute of Brain Science, National Yang Ming Chiao Tung University, Taipei, Taiwan;

[10] Division of Psychiatry, Faculty of Medicine, National Yang Ming Chiao Tung University, Taipei, Taiwan;

[11] Membrane Protein Structural Biology Research Center, National Yang Ming Chiao Tung University, Taipei, Taiwan;

[12] Ph.D. Program in Medical Neuroscience, College of Medical Science and Technology, Taipei Medical University and National Health Research Institute, Taipei, Taiwan

*Address for reprint requests and correspondence:

Cheng-Ta Li MD. PhD, Department of Psychiatry, Taipei Veterans General Hospital, Taipei, Taiwan. No. 201., Sec. 2, Shih-Pai Road, Taipei 112, Taiwan.

E-mail: on5083@msn.com

Hui-Ching Lin. PhD, Department and Institute of Physiology, College of Medicine, National Yang Ming Chiao Tung University, Taipei 11221, Taiwan; Phone: +886-2-2826-7944; Fax: +886-2-2826-4049.

E-mail: hclin7@nycu.edu.tw; huiching4372@gmail.com

Supplemental Materials and Methods

Western blot assay

The PFC tissues were carefully dissected and homogenized in lysis buffer composed of 1% Triton X-100, 0.1% SDS, 50 mM Tris-HCl (pH 7.5), 0.3 M sucrose, 5 mM EDTA, 2 mM sodium pyrophosphate, 1 mM sodium orthovanadate, and 1 mM phenylmethylsulfonyl fluoride, supplemented with a complete protease inhibitor cocktail. After sonication, the homogenates were centrifuged at 12,000 rpm for 30 minutes to separate the supernatants. Protein concentrations were determined using the Bradford assay, and equal amounts of protein were loaded onto SDS-PAGE gels for electrophoresis. Proteins were then transferred to Immobilon-P membranes (Millipore) and blocked with 5% nonfat dry milk for 1 hour at room temperature. Western blotting was performed using the following primary antibodies: phosphorylation of STAT3 at Tyr705 (Cell Signaling Technology), STAT3 (Cell Signaling Technology), phosphorylation of GSK3β at Ser9 (Cell Signaling Technology), GSK3β (Cell Signaling Technology) ,GluA1 (Abcam), GluA2 (Millipore), PSD-95 (Cell Signaling Technology), and β-actin (Abcam), GAPDH (GeneTex). Membranes were incubated with the primary antibodies overnight at 4°C, followed by HRP-conjugated secondary antibody incubation for 1 hour at room temperature. Protein signals were visualized using ECL Plus detection reagent (PerkinElmer, Boston, MA). Films were exposed for varying durations to ensure accurate signal detection without saturation, and band intensities were quantified using densitometry. The relative optical densities of the protein bands were analyzed with ImageJ software. For quantification, protein levels were first normalized to internal control and then expressed as fold changes relative to non-CSDS samples.

Statistical analysis

Data were analyzed using GraphPad Prism version 6 (GraphPad Software, San Diego, CA, USA). Results are presented as mean ± standard error of the mean (SEM), with SEM values shown as percentages. To assess group differences, Kruskal–Wallis test were employed. This statistical approach was applied to evaluate behavioral performance, electrophysiological data, dendritic spine density, and protein expression levels between non-CSDS and susceptible groups. A p-value of less than 0.05 was considered statistically significant.

Supplemental Results

Supplemental figure 1.

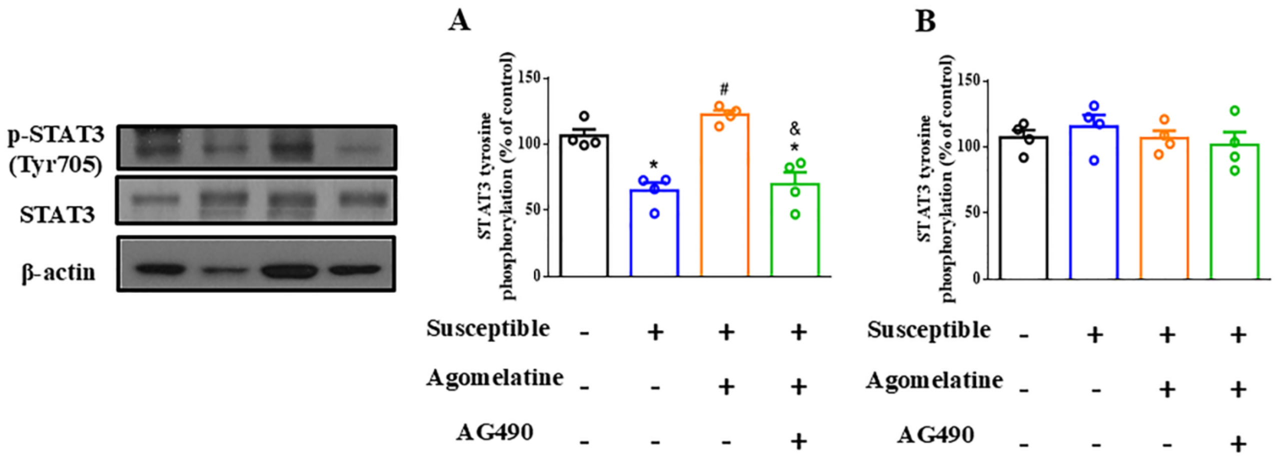

Supplementary Figure 1. The STAT3 phosphorylation and expression in PFC of susceptible mice treating with agomelatine and combine with AG490.

(A) Representative Western blot and summary bar graph of phosphorylation levels of STAT3 in the PFC. (B) Representative Western blot and summary bar graph of expression levels of STAT3 in the PFC. Data are represented as the mean ± SEM in each experiment. *$p < 0.05$ versus non-CSDS group, #$p < 0.05$ versus susceptible group, &$p < 0.05$ versus agomelatine treated group by Kruskal–Wallis test (each group n = 4).

Supplemental figure 2.

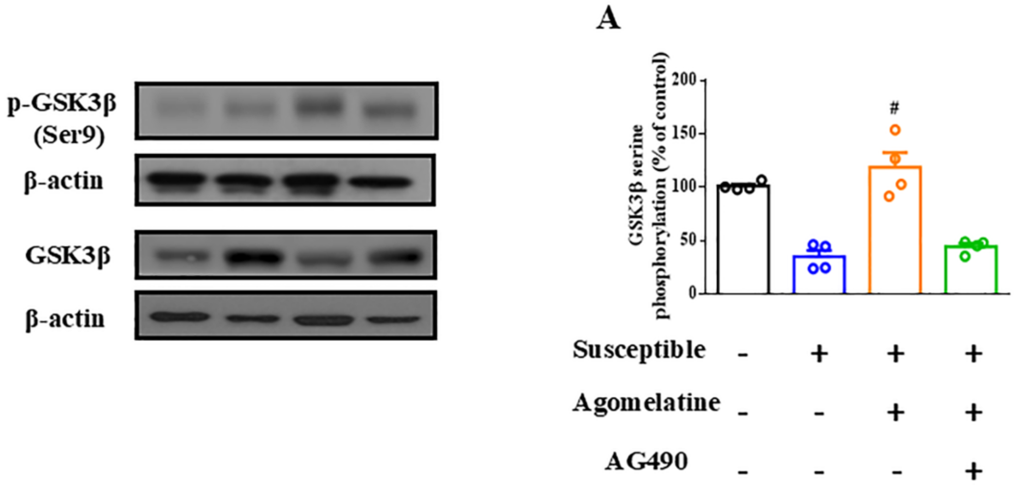

Supplementary Figure 2. The GSK3β phosphorylation and expression in PFC of susceptible mice treating with agomelatine and combine with AG490.

(A) Representative Western blot and summary bar graph of phosphorylation levels of GSK3β in the PFC, with phosphorylated and total GSK3β detected on separate membranes and each normalized to its respective housekeeping control (β-actin). Data are represented as the mean ± SEM in each experiment. #$p < 0.05$ versus susceptible group by Kruskal–Wallis test (each group n = 4).
